# Gut Microbiota Modulation in IBD: From the Old Paradigm to Revolutionary Tools

**DOI:** 10.3390/ijms26073059

**Published:** 2025-03-27

**Authors:** Marco Murgiano, Bianca Bartocci, Pierluigi Puca, Federica di Vincenzo, Angelo Del Gaudio, Alfredo Papa, Giovanni Cammarota, Antonio Gasbarrini, Franco Scaldaferri, Loris Riccardo Lopetuso

**Affiliations:** 1 Dipartimento di Medicina e Chirurgia Traslazionale, Università Cattolica del Sacro Cuore, 00168 Rome, Italy; biancabartocci97@gmail.com (B.B.); pgpuca@gmail.com (P.P.); federica.divincenzo30@gmail.com (F.d.V.); delgaudioangelo@gmail.com (A.D.G.); alfredo.papa@policlinicogemelli.it (A.P.); giovanni.cammarota@policlinicogemelli.it (G.C.); antonio.gasbarrini@policlinicogemelli.it (A.G.); franco.scaldaferri@policlinicogemelli.it (F.S.); 2 Medicina Interna e Gastroenterologia, CEMAD Centro Malattie dell’Apparato Digerente, Dipartimento di Scienze Mediche e Chirurgiche, Fondazione Policlinico Universitario Gemelli IRCCS, 00168 Rome, Italy; 3 Dipartimento di Scienze della Vita, della Salute e delle Professioni Sanitarie, Università degli Studi Link, 00165 Rome, Italy

**Keywords:** gut microbiota, dysbiosis, inflammatory bowel disease, probiotics, fecal microbiota transplantation, microbiota modulation, microbial biotherapeutics

## Abstract

Inflammatory bowel diseases (IBDs) are chronic inflammatory disorders primarily comprising two main conditions: ulcerative colitis and Crohn’s disease. The gut microbiota’s role in driving inflammation in IBD has garnered significant attention, yet the precise mechanisms through which the microbiota influences IBD pathogenesis remain largely unclear. Given the limited therapeutic options for IBD, alternative microbiota-targeted therapies—including prebiotics, probiotics, postbiotics, and symbiotics—have been proposed. While these approaches have shown promising results, microbiota modulation is still mainly considered an adjunct therapy to conventional treatments, with a demonstrated impact on patients’ quality of life. Fecal microbiota transplantation (FMT), already approved for treating *Clostridioides difficile* infection, represents the first in a series of innovative microbiota-based therapies under investigation. Microbial biotherapeutics are emerging as personalized and cutting-edge tools for IBD management, encompassing next-generation probiotics, bacterial consortia, bacteriophages, engineered probiotics, direct metabolic pathway modulation, and nanotherapeutics. This review explores microbial modulation as a therapeutic strategy for IBDs, highlighting current approaches and examining promising tools under development to better understand their potential clinical applications in managing intestinal inflammatory disorders.

## 1. Introduction

Inflammatory bowel diseases (IBDs) are chronic inflammatory disorders of the gastrointestinal tract characterized by a relapsing-remitting course, primarily including Crohn’s disease (CD) and ulcerative colitis (UC) [1]. Although the exact etiology of IBD remains unknown, the most widely accepted hypothesis suggests that IBD results from an inappropriate immune response triggered by a combination of genetic, microbial, and environmental factors [2]. Despite its multifactorial nature, microbial dysfunction is recognized as one of the key contributors to IBD pathogenesis [3].

The gut microbiome is a complex ecosystem that maintains a mutualistic relationship with the host, playing a crucial role in preserving homeostasis [4]. Dysbiosis refers to any disruption in the gut microbiota composition that disturbs microbial balance, ultimately impairing gut functions and disrupting host–microbiota homeostasis [5,6]. Many studies have identified dysbiosis as a significant factor contributing to both the pathogenesis and progression of IBD [7,8].

Inflammatory bowel diseases (IBDs) arise from a dysregulated immune response to an altered microbiota in genetically predisposed individuals, with environmental factors acting as triggers [9]. Understanding the dynamic interplay between host genetics and the gut microbiota is challenging due to the large number of genomic markers and the diversity of microbial taxa. Genetic polymorphisms can affect immune responses or gut barrier function, leading to impaired bacterial clearance and establishing a link between genetic variants and gut dysbiosis. Many studies have shown similarities in the gut microbiota of twins, regardless of their concordance or discordance for IBD, highlighting the genetic influence on microbiota composition [10,11].

Currently, around 240 risk loci associated with IBD have been identified, with some of the most well-documented being *NOD2*, *ATG16L1*, *IGRM*, and *CARD9* [12,13,14]. For instance, associations between *NOD2* and *ATG16L1* genotypes and dysbiosis have been observed in Crohn’s disease patients [15]. Specific variants of the *NOD2* gene have been linked to increased abundance of the Enterobacteriaceae family in IBD patients [16]. Moreover, genetic variations can influence gut-derived metabolites, such as alterations in the *MYRF* gene, which are associated with changes in short-chain fatty acids (SCFAs)—key molecules for maintaining gut health [17].

In recent years, increasing attention has been given to microbial genome sequencing and host–microbe interactions to advance targeted IBD therapies. Integrative multiomics studies have characterized IBD patients by analyzing host genetics, microbiota, and other risk factors. For instance, a recent case-control study documented gut microbiota alterations (e.g., decreased *Roseburia* abundance) in healthy individuals with a high genetic risk for IBD [18].

Beyond genetic factors, environmental exposures such as diet, antibiotic use, infections, and smoking can disrupt the microbial balance, further implicating the microbiota in IBD pathogenesis [19,20]. Smoking influences both microbiota composition and immune responses [21] and can impair intestinal barrier function with chronic exposure [22]. The Western diet, rich in saturated fats and refined carbohydrates but low in fiber, is considered a trigger for IBD due to its impact on microbiota diversity, mucosal integrity, and immunity [23]. This diet promotes the proliferation of mucin-degrading bacteria like *Bacteroides thetaiotaomicron* and *Akkermansia muciniphila*, as well as potentially pathogenic species like *Proteobacteria* and adherent-invasive *E. coli*, ultimately leading to barrier dysfunction [24,25,26]. In contrast, a fiber-rich plant-based diet promotes microbial diversity and increases the production of SCFAs, which help maintain intestinal health [27].

Early-life antibiotic exposure can also shape gut microbiota composition, with different effects depending on the antibiotic type and duration of use. A recent meta-analysis linked exposure to cephalosporins, quinolones, tetracyclines, and metronidazole with an increased risk of developing IBD, particularly with earlier antibiotic exposure [28,29].

The gut–brain axis adds another layer of complexity to IBD. Stress, depression, and anxiety can alter microbiota composition and gut-derived metabolites, exacerbating inflammation, while gut microbiota modulation can influence stress responses [30,31]. Fecal microbiome richness and diversity are lower in IBD patients with psychological disorders than in those without, with a reduction in beneficial bacteria (e.g., *Lachnospiraceae*, *Fusobacteriaceae*, *Ruminococcaceae*, and *Veillonellaceae*) and an increase in opportunistic pathogens like *Enterococcus* [32,33,34]. Notably, reduced abundance of *Roseburia*, a butyrate-producing bacterium, has been linked to depression in Crohn’s disease [33].

Together, genetic and environmental factors influence not only disease pathogenesis but also therapeutic responses. It remains unclear whether gut microbiota alterations are a cause or consequence of intestinal inflammation, but unraveling this relationship could pave the way for innovative therapeutic strategies [35,36].

Despite advancements in understanding IBD, current therapies—including 5-aminosalicylates (5-ASA), corticosteroids, biologics, and small molecules—have limitations, such as adverse effects, drug resistance, and variable responsiveness. This has driven interest in therapies that modulate the gut microbiota to influence disease outcomes. Probiotics, for example, have demonstrated safety and efficacy in preventing disease flares and improving quality of life, with minimal contraindications in IBD patients [37,38].

Fecal microbiota transplantation (FMT) was the first microbiota-targeted therapy, showing promise in treating not only IBD but also conditions like chronic liver disorders and extraintestinal autoimmune diseases [39]. Meanwhile, preclinical studies on microbial biotherapeutics—including next-generation probiotics, bacterial consortia, bacteriophage-based therapies, engineered probiotics, direct metabolic pathway modulation, and nanotherapeutics—have yielded encouraging, albeit preliminary, results.

## 2. Changes in Gut Microbiota Are Associated with IBD

Gut microbiota is a dynamic community composed of a vast array of commensal bacterial species [40]. More than 90% of the human gut microbiota belongs to four main phyla: *Firmicutes*, *Bacteroidetes*, *Actinobacteria*, and *Proteobacteria* [41,42]. The relative abundance of these phyla, which reflects microbial diversity, can vary significantly between individuals [43]. Two essential concepts in understanding intestinal health are eubiosis and dysbiosis, representing opposite states of microbial balance with distinct effects on human health. Eubiosis refers to a state of microbial equilibrium, characterized by a predominance of beneficial bacteria that contribute to overall intestinal well-being [44].

In a healthy gut, the microbiota and host maintain a symbiotic relationship that strengthens immune system function, aids digestion and nutrient absorption, and reduces the risk of developing inflammatory bowel disease (IBD) [45]. Conversely, dysbiosis refers to an imbalance in the composition and function of the gut microbiota, disrupting the microbe–host relationship and impairing critical physiological functions [5].

Many studies have identified dysbiosis as a central factor in IBD pathogenesis [7,8]. Several mechanisms explain this connection. A hallmark of dysbiosis in IBD patients is the depletion of butyrate-producing bacteria, alongside an increase in sulfate-reducing bacteria. The loss of short-chain fatty acid (SCFA)-producing species, such as *Faecalibacterium prausnitzii*, weakens the intestinal epithelial barrier, increasing gut permeability and promoting bacterial translocation across the intestinal epithelium and into the lamina propria [46]. This also impairs the differentiation of regulatory T cells (Tregs), which are essential for immune tolerance [47].

Additionally, an overgrowth of lipopolysaccharide (LPS)-producing bacteria can trigger toll-like receptor 4 (TLR4) signaling, activating NF-κB and perpetuating chronic inflammation [48]. The expansion of sulfate-reducing bacteria exacerbates the condition by converting sulfate to hydrogen sulfide, a compound that inhibits butyrate utilization, impairs immune responses, and facilitates bacterial persistence [49].

Oxidative stress is another driver of dysbiosis and IBD. The increased production of reactive oxygen species (ROS) disrupts the gut’s anaerobic environment, fostering the growth of facultative anaerobes and furthering microbial imbalance [50].

Bile acid metabolism is also intricately linked to dysbiosis and IBD. Alterations in the gut microbiota can disturb bile acid homeostasis, reducing secondary bile acid production and impairing farnesoid X receptor (FXR) signaling, a key regulator of bile acid metabolism [51]. Similarly, disruptions in tryptophan metabolism limit the generation of microbial-derived aryl hydrocarbon receptor (AhR) ligands, compromising intestinal barrier integrity and promoting immune dysregulation [52].

Together, these mechanisms position dysbiosis as an active driver of IBD, emphasizing the potential of microbiome-targeted therapeutic strategies. Interventions such as dietary modifications, metabolite-based therapies, and fecal microbiota transplantation offer promising avenues for restoring microbial balance and mitigating IBD severity.

The composition of the gut microbiota differs significantly between IBD patients and healthy controls [53]. Individuals with IBD exhibit distinct alterations in microbial abundance, alongside reduced α- and β-diversity of intestinal flora [54]. At the phylum level, IBD patients typically show a decrease in *Firmicutes*—commensal bacteria essential for maintaining the intestinal barrier and mucosal immunity [55,56]—while displaying an increased abundance of *Proteobacteria* and *Bacteroidetes*, both associated with pro-inflammatory properties [55,57].

At the family level, a reduction in beneficial, anti-inflammatory bacteria, such as *Lachnospiraceae* and *Ruminococcaceae*, which play a crucial role in short-chain fatty acid (SCFA) production, has been observed in IBD patients compared to healthy controls [58,59]. At the genus level, lower levels of *Roseburia*, *Akkermansia*, and *Faecalibacterium* have been documented [60,61,62]. Additionally, IBD patients show decreased levels of beneficial bacteria like *Bacteroides*, *Lactobacillus*, and *Eubacterium* [63,64], alongside an increase in potentially harmful species such as *Enterobacteriaceae*, *Pasteurellaceae*, *Veillonellaceae*, and *Fusobacteriaceae* [3,65,66,67,68].

These microbial changes are not only associated with the presence of IBD but also correlate with disease activity. A meta-analysis of 16 studies, including 1669 subjects, demonstrated a significantly lower abundance of *F. prausnitzii* in IBD patients compared to healthy controls. Furthermore, *F. prausnitzii* levels were lower in patients with active IBD (both UC and CD) compared to those in remission, suggesting a negative correlation between *F. prausnitzii* abundance and disease activity [69].

Interestingly, shifts in microbiota composition have also been observed following treatment. For example, anti-TNF-α therapy has been associated with a significant increase in *F. prausnitzii* abundance [70]. Higher fecal levels of *F. prausnitzii* have also been linked to lower Crohn’s Disease Activity Index (CDAI) scores, C-reactive protein (CRP) levels, and erythrocyte sedimentation rates [71]. Given the extreme sensitivity of *F. prausnitzii* to changes in the intestinal environment, its detection in feces or mucosa may serve as a valuable biomarker for diagnosing IBD and monitoring disease prognosis [72].

The rise of integrated multiomics approaches now enables a more comprehensive analysis of the relationship between gut dysbiosis and IBD, characterizing bacterial, fungal, and metabolic fingerprints associated with the disease [73]. Considering the well-established link between gut microbiota alterations and IBD, ongoing research is increasingly focused on developing innovative microbiome-targeted therapeutic strategies.

## 3. The Old Paradigm of Gut Microbiota Modulation in IBD

Despite the wide range of therapeutic options available for UC and CD, including anti-inflammatory and immunosuppressive drugs, treatment efficacy remains limited, recurrence rates are high, and symptom control is often inadequate. As a result, alternative therapeutic strategies have been developed to improve IBD management.

### 3.1. Antibiotics

Several antibiotics, including metronidazole, ciprofloxacin (alone or in combination), rifaximin, and antitubercular therapy, have been evaluated in multiple trials for the treatment of IBD [74]. A meta-analysis of 10 randomized trials reported improved clinical outcomes in IBD patients following antibiotic therapy (ciprofloxacin, metronidazole, and clarithromycin, either individually or combined) [75]. In CD, antibiotics serve various purposes: metronidazole has been used in colonic Crohn’s disease [76], while ciprofloxacin has been compared to mesalamine in active luminal disease [76]. Complications like abscesses and fistulas often require drainage and antibiotic therapy (ciprofloxacin, metronidazole, or both) [77], and antibiotics also play a role in preventing post-operative recurrence [78]. Conversely, evidence supporting antibiotic use in UC is more limited [79]. Antibiotics, particularly metronidazole and ciprofloxacin, remain the cornerstone of pouchitis treatment [80]. Other indications include small intestinal bacterial overgrowth, septic complications, or perianal disease.

Despite their potential benefits, the use of antibiotics in IBD remains controversial due to adverse effects, the risk of dysbiosis, increased susceptibility to *C. difficile* infections, and the emergence of antibiotic-resistant strains [57,81]. For example, rifaximin, long considered a safe option, has recently been linked to resistance development. Rifaximin prophylaxis has been associated with daptomycin resistance—a last-resort therapy for multidrug-resistant pathogens like vancomycin-resistant *Enterococcus faecium* (VREfm)—especially in patients with liver cirrhosis [82]. These findings emphasize the importance of judicious antibiotic use, even with ‘low-risk’ agents, as they may contribute to antimicrobial cross-resistance. Antibiotic resistance is a global crisis, driven by overuse in both the general population and healthcare settings. In IBD patients, especially in scenarios like bowel obstruction, the indication for antibiotics remains unclear due to the elevated risk of *C. difficile* infection.

### 3.2. Probiotics

Probiotics are live microorganisms that exhibit antibacterial properties, counteracting pathogenic bacteria, modulating gut microbiota composition, regulating the immune system, enhancing anti-inflammatory responses, and strengthening the intestinal barrier [83,84,85]. Several studies have shown that administering specific probiotic strains, such as *E. coli* Nissle 1917, *Bifidobacteria*, and *Lactobacillus*, can regulate and modulate immune responses. Probiotics influence immune activity by modulating the functions of macrophages, dendritic cells, and T lymphocytes [86]. Probiotics have been studied not only for IBD but also for other conditions, such as irritable bowel syndrome and diverticulitis. For instance, various *Lactobacilli* strains (e.g., *Limosilactobacillus reuteri* ATCC PTA 4659) have shown anti-inflammatory effects, reducing serum and fecal inflammatory markers in acute uncomplicated diverticulitis [87]. The administration of *Clostridium butyricum* has been associated with improved symptoms, including increased stool consistency and reduced urinary urgency in irritable bowel syndrome [88]. The most significant benefits of probiotics are observed in UC patients (both in active disease and remission), while data regarding their efficacy in CD remain inconsistent [89,90]. Recent studies on the effectiveness of probiotics are summarized in Table 1.

Although probiotics are widely used to prevent and treat various gastrointestinal (GI) diseases, several factors must be carefully considered: the mechanisms of action of each strain, the optimal dosage, the timing and duration of treatment, the selection of individual strains or their combinations, as well as safety and stability. Therefore, large-scale randomized controlled trials are essential to confirm the efficacy of probiotics and establish their role in clinical practice.

### 3.3. Prebiotics

Prebiotics are nondigestible (oligo)saccharides that confer physiological benefits to the host by selectively promoting the growth or activity of a limited number of commensal bacteria [102]. They must undergo selective fermentation by specific beneficial bacteria in the intestine. The most commonly used prebiotics are β-fructans, oligosaccharides, and inulin [103,104].

The effects of prebiotics have been primarily investigated in experimental colitis models. Their role in maintaining intestinal barrier integrity by fostering the growth of bacteria that upregulate defense mechanisms has been documented [105]. Prebiotics can also influence the production of pro- and anti-inflammatory cytokines, mitigating the inflammatory response [106]. Lactulose and inulin have demonstrated promising anti-inflammatory properties [107]. In a dextran sulfate sodium (DSS)-induced mouse model, administering fructo-oligosaccharide (400 mg/kg) for 37 days alleviated DSS-induced colitis symptoms by modulating intestinal dysbiosis and tryptophan metabolism [108]. Fructo-oligosaccharide also exhibits anti-inflammatory effects in T lymphocyte-dependent colitis models by reducing pro-inflammatory cytokine levels [109].

The effects of prebiotics in human studies remain under investigation. In a small open-label trial involving ten patients with ileocolic Crohn’s disease, supplementation with 15 g/day of oligofructose and inulin for 3 weeks reduced clinical disease activity, accompanied by an increase in mucosal *Bifidobacteria* [110]. This finding was corroborated by subsequent studies showing that administering 12 g/day of oligofructose-enriched inulin reduced quantitative fecal calprotectin levels [111]. Recent research has highlighted the potential of prebiotics in reducing clinical and endoscopic disease activity. In a single-center, randomized, double-blind, placebo-controlled trial, 9.8 g/day of trisaccharide fructooligosaccharides-1 ketose led to higher clinical remission and response rates (with no significant difference in endoscopic outcomes) and a significant reduction in α-diversity in patients with mild to moderate UC [112]. An improvement in endoscopic activity was observed in a dual-arm exploratory study involving the administration of either 15 g/day or 7.5 g/day of β-fructans inulin plus fructooligosaccharides in patients with mild to moderate active UC [113].

Despite these promising results, further human studies are necessary to confirm the true effectiveness of prebiotics in IBD management.

### 3.4. Postbiotics

Postbiotics are inactivated microbial cells or their components, with or without metabolites, that provide health benefits. They play a crucial role in the development of the intestinal immune system and the maintenance of the intestinal barrier, while also indirectly shaping microbiota composition [114].

Interest in postbiotics for IBD treatment is increasing, with several studies highlighting their potential benefits for patients. For instance, microencapsulated sodium butyrate, known for its anti-inflammatory and regenerative properties, has been shown to induce microbiota changes in IBD patients. In UC patients, it promoted the growth of beneficial short-chain fatty acid-producing bacteria, such as *Lachnospiraceae* spp., while in CD patients, it increased the abundance of butyrogenic colonic bacteria, including *Butyricicoccus* [115]. Additionally, the administration of SER-287 (*Firmicutes* spores) after vancomycin preconditioning demonstrated efficacy in achieving clinical remission in patients with mild to moderately active UC [116].

Regulatory guidelines require a precise limit on the number of viable microorganisms remaining after postbiotic preparation. Further exploration of postbiotics may open new frontiers in IBD treatment, offering a dual benefit: maintaining homeostasis and modulating the intestinal barrier to restore eubiosis.

### 3.5. Symbiotics

Beneficial effects in IBD can also arise from symbiotics, a combination of probiotics and prebiotics. The most well-known and widely used combinations include *Bifidobacteria* with fructooligosaccharides, *Bifidobacteria* and *Lactobacilli* with fructooligosaccharides or inulin, and *Lactobacillus* GG with inulin. Several trials have demonstrated the efficacy of symbiotic therapy in IBD [117,118]. For example, supplementation with symbiotics (*Bifidobacterium longum* combined with inulin-oligofructose or psyllium) in UC patients showed greater clinical improvement compared to probiotics or prebiotics alone [119]. Additionally, the use of *B. longum*, inulin, and oligofructose in UC patients reduced endoscopic inflammation and serum markers of inflammation, including TNF-α and IL-1β [119]. Similarly, the combination of *L. rhamnosus 1.0320* with inulin alleviated DSS-induced colitis by enhancing gut flora diversity and increasing the abundance of beneficial bacteria [120].

More recently, a symbiotic combination of *Lactobacillus plantarum SC-5* and olive oil extract tyrosol in a DSS-induced mice model led to colitis remission and reduced colonic inflammation by inhibiting mitogen-activated protein kinase (MAPK) and NF-κB pathways. The combination also preserved gut barrier integrity by regulating the expression of occludin, claudin-3, and zona occludens-1 [121].

Although symbiotics have demonstrated greater effectiveness than probiotics or prebiotics alone, their precise clinical impact remains unclear due to variability in benefits depending on the specific probiotic–prebiotic combinations. Existing studies often use heterogeneous methodologies and suffer from limitations in study design. Moreover, many findings come from preclinical models, limiting their immediate application to clinical practice. The optimal dosage and duration of treatment also remain undefined.

## 4. Revolutionizing Microbiota Therapeutics in IBD: The New Paradigm

More recently, building on the encouraging results from studies on probiotics, prebiotics, symbiotics, and postbiotics, research has shifted towards exploring even more innovative experimental therapeutic approaches. The modulation of gut microbiota remains the underlying rationale for these strategies. However, the goal is to develop more effective and targeted tools for clinical practice—with the ambitious aims of increasing response rates, minimizing the impact on microbial resistance, and ensuring an excellent safety profile. 

### 4.1. Fecal Microbiota Transplantation

Fecal microbiota transplantation (FMT) is emerging as a promising therapeutic tool with a wide range of potential applications. Its primary goal is to restore the natural composition of intestinal flora by transferring fecal microbiota from healthy donors [122]. Various administration methods are available, including nasogastric or nasojejunal tubes, colonoscopy, enema, and oral capsules [123,124]. Currently, the most established use of FMT is for treating antibiotic-resistant *C. difficile* infections [125]. A randomized controlled trial reported clinical improvement, with diarrhea resolution in 81% of patients receiving FMT, compared to only 31% in the antibiotic-treated group [126].

Many studies have evaluated FMT’s efficacy in ulcerative colitis (UC), exploring different delivery routes and infusion schedules, though results have been inconsistent: they are summarized in Table 2 [127,128,129,130,131,132,133,134,135]. A 2023 meta-analysis of 13 randomized controlled trials, including 580 UC patients (293 receiving FMT and 287 controls), showed significantly higher clinical and endoscopic remission rates at 8–12 weeks in the FMT group, suggesting its potential to induce UC remission [136]. Interestingly, clinical remission and response rates were consistent across trials, despite methodological heterogeneity.

Another meta-analysis, including 9 studies with 520 UC patients (261 receiving FMT and 259 controls), found significantly improved clinical remission [RR = 1.53; 95% CI: 1.19–1.94; *p* < 0.0008] and endoscopic remission [RR = 2.80; 95% CI: 1.93–4.05; *p* < 0.00001] in the FMT group. Administration routes included colonoscopy, enema, and nasoduodenal tubes, with follow-ups ranging from 7 to 48 weeks, though remission differences beyond 8 weeks were not statistically significant, emphasizing the need for long-term studies [137].

Long-term FMT efficacy in UC remains underexplored. In a randomized, double-blind, placebo-controlled trial, Haifer et al. followed UC patients on oral lyophilized FMT. At week 8, responders were randomized to continue or discontinue FMT for an additional 48 weeks. By week 56, all patients continuing FMT (4/4) maintained remission, while none in the discontinuation group (0/6) remained in remission [128]. Similarly, Costello et al. tracked UC patients for 12 months after an 8-week FMT induction via colonoscopy and enema, finding that 42% (5/12) maintained remission at 12 months [131]. These findings highlight FMT’s potential but underscore the need for larger, long-term trials.

FMT research in Crohn’s disease (CD) is more limited, often lacking control groups and featuring small sample sizes, leading to mixed outcomes [138]. Methodological variability—such as FMT type, delivery routes, antibiotic preconditioning, infusion frequency, and donor selection—further complicates interpretation. A meta-analysis of 12 studies (1 RCT, 7 cohort studies, 4 case reports) showed clinical remission and response rates of 62% and 79%, respectively, with follow-ups from 4 weeks to 15 months. Remission correlated with increased microbiota diversity and stability [139]. A systematic review of 11 cohort studies and 1 RCT supported these findings, showing short-term clinical remission, improved disease activity indices, and enhanced microbial diversity [140]. Notably, multiple FMT infusions yielded higher response rates than single infusions [141].

For chronic pouchitis, evidence is sparse. A meta-analysis of 8 studies (74 receiving FMT, 15 placebo) showed short-term clinical responses in 42% of patients [142]. Another analysis of 7 cohort studies and 2 RCTs (103 patients) reported a clinical response rate of 42.6% and remission in 29.8%, though delivery methods varied widely—ranging from upper GI tract routes (nasogastric tube, jejunal endoscopy) to lower GI tract options (pouchoscopy, enemas) [143].

An intriguing aspect of FMT is the potential transfer of not only bacteria but also viruses. Although bacteria constitute the dominant gut microbiota, viruses can interact with the host, influence immune responses, and affect metabolic functions [144].

Despite its promise, FMT poses challenges. Most studies have short follow-ups, so long-term safety and efficacy data are lacking. Donor variability introduces qualitative and quantitative microbiota differences, affecting outcomes [145]. The transfer of live microorganisms to immunocompromised patients carries inherent risks, though rigorous donor screening can mitigate this [146]. Donor selection is complex and costly, requiring extensive clinical and microbiological screening to minimize pathogen transmission. Additionally, standardizing fecal preparations is difficult, as microbiota composition varies with diet, lifestyle, and antibiotic history, influencing treatment effectiveness and remission rates. The logistical costs of biological material handling and storage further complicate clinical implementation [147].

Given these complexities, patient and donor selection must be meticulous. While FMT holds great potential for managing IBD and other conditions, addressing current limitations through robust, long-term research will be essential to fully unlock its clinical benefits.

### 4.2. Rising Star Probiotics: Faecalibacterium Prausnitzii

Recently, live biotherapeutic products have emerged as a promising new treatment. These products are defined as commensal strains isolated from healthy donors, subsequently cultured, characterized, and carefully selected based on their anti-inflammatory and biological effects. One notable example is the anti-inflammatory strain of *F. prausnitzii*, which constitutes 3–5% of the total bacterial population and is one of the most predominant bacterial groups in human feces [148].

It has been proposed that *Faecalibacterium* plays a key role in stabilizing gut microbiota [149]. This bacterium exerts anti-inflammatory effects by producing molecules that target the IL-1β-induced NF-κB pathway, thereby reducing IL-8 secretion by intestinal epithelial cells [150]. Additionally, *F. prausnitzii* promotes the production of anti-inflammatory cytokines while inhibiting the secretion of pro-inflammatory cytokines such as IFN-γ, TNF-α, IL-6, and IL-12 [151].

Beyond its immunomodulatory properties, *F. prausnitzii* generates metabolites that enhance intestinal barrier function and regulate gut permeability by increasing the expression of tight junction proteins like E-cadherin [152]. It is also considered one of the primary intestinal producers of butyrate, a short-chain fatty acid (SCFA) with potent anti-inflammatory effects [153,154]. Through these mechanisms, *F. prausnitzii* contributes to maintaining host homeostasis, limiting intestinal bacterial translocation, and reducing inflammation—making it a highly relevant candidate for innovative IBD therapies [155].

Notably, a reduction in *Faecalibacterium* abundance has been observed in IBD patients, with a significant inverse correlation between *F. prausnitzii* levels and disease activity in UC, further highlighting the bacterium’s protective and anti-inflammatory effects [156]. These findings have been validated in moderate and severe DNBS-induced chronic colitis mouse models. Colitis was induced via intrarectal DNBS administration, and after a 4-day recovery period (for moderate colitis) or 10 days (for severe colitis), intragastric *F. prausnitzii* A2-165 was administered for 7 or 10 days. When colitis was reactivated with a lower DNBS dose, *F. prausnitzii* treatment significantly reduced disease severity in both models [157].

### 4.3. Rising Star Probiotics: Akkermansia Muciniphila

Another promising probiotic is *Akkermansia muciniphila* (*A. muciniphila*), one of the most abundant species in the intestinal microbiota [158]. It plays a crucial role in maintaining intestinal health, regulating metabolism, and modulating immune responses [159]. Many studies have demonstrated its ability to reduce macrophage and CD8 lymphocyte infiltration, thereby alleviating colitis and increasing the number of anti-inflammatory T-reg cells [160,161]. Its role in metabolic diseases such as obesity, diabetes, and alcoholic liver disease is well established [162,163,164], although its function in IBD remains less clear.

Different administration methods for *A. muciniphila* have been explored, including viable, pasteurized forms, and through specific components such as AmEVs, Amuc_1100, and P9 [165]. Reduced levels of *A. muciniphila* have been observed in patients with active UC or CD [68,166,167], with a significant decrease in active UC patients compared to those with quiescent UC and healthy controls [168]. Furthermore, the lower density of *A. muciniphila* in active UC patients has been confirmed, suggesting that its levels may serve as a marker for disease exacerbation [169]. In animal models, oral administration of *A. muciniphila* in DSS-induced colitis reduced CD8+ lymphocytes and macrophages, effectively limiting inflammation [170].

Despite promising findings, the roles of *A. muciniphila* and *F. prausnitzii* in IBD are not yet fully understood. While next-generation probiotics (NGPs) offer potential therapeutic benefits, concerns remain regarding their safety, dosage, and efficacy. Safety is a critical factor in NGP development [171], as long-term exposure to probiotics may alter microbial functions or negatively impact the surrounding microbiota [172]. Additionally, disruption of the intestinal barrier could allow probiotics to enter systemic circulation, increasing the risk of invasive infections [173].

Next-generation probiotics are being designed to be more resilient, targeted, and personalized to individual microbiome compositions, enhancing their therapeutic potential. Although preclinical studies and early-phase clinical trials show encouraging results, larger and more rigorous studies are necessary to confirm the efficacy of NGPs for treating chronic diseases [174]. Regulatory approval remains a major challenge, requiring extensive research to assess long-term safety and efficacy. For this reason, ethical approval is essential for larger sample sizes and long-term evaluations [174].

The development of new probiotics requires complete genome sequencing to characterize bacterial strains and their physiological traits, including antibiotic resistance and growth dynamics [175]. However, little is known about the safety profiles of *Faecalibacterium* and *Akkermansia*.

*F. prausnitzii* is an obligate anaeorobe, thriving only in oxygen-free environments.

Its extreme oxygen sensitivity makes cultivation, stabilization, growth, and production of a viable oral probiotic particularly challenging [176]. Even brief exposure to air can rapidly kill the bacteria, complicating manufacturing, packaging, and delivery processes for human use [148]. Its biodiversity and physiological characteristics remain unclear, and genome analysis suggests potential resistance to multiple antibiotics [177]. For example, Machado et al. found that *F. prausnitzii* DSM17677 exhibited phenotypic resistance to ampicillin, gentamicin, kanamycin, and streptomycin [178], while Hu et al. showed susceptibility to vancomycin but resistance to kanamycin and gentamicin [179]. Further studies are needed to understand the mechanisms underlying antibiotic resistance in *F. prausnitzii* and its clinical implications.

Although *A. muciniphila* is also challenging to culture, more oral formulations are available, making it a more promising candidate for clinical use [180]. However, its extreme oxygen sensitivity complicates isolation and purification. Since only a single strain of *A. muciniphila* is widely available for research, analyzing resistance patterns across different strains is difficult. Experiments have shown that *A. muciniphila* is susceptible to imipenem and doxycycline but resistant to metronidazole and vancomycin [181]. Strain-specific variations in antibiotic resistance have also been observed. For example, *A. muciniphila* D22959 exhibited different susceptibilities to gentamicin and kanamycin [181,182]. These findings underscore the need to establish standardized protocols for accurately assessing the antibiotic sensitivity of *A. muciniphila* strains.

Determining the correct NGP dosage for beneficial effects in humans remains a challenge [183]. Additional research is also needed to explore the possibility of administering multiple NGPs concurrently and to investigate potential synergistic effects [184]. With rigorous clinical trials and strong regulatory support, next-generation probiotics could become a cornerstone of personalized medicine, providing targeted therapies for chronic diseases and improving overall health outcomes.

### 4.4. Bacterial Consortia

An innovative and promising therapeutic strategy for microbiota modulation involves the use of bacterial consortia to regulate gut microbiota commensals. A microbial consortium consists of two or more symbiotic microorganisms capable of surviving under various conditions by forming synergistic population structures such as stromatolites, microbial mats, and biofilms [185].

The concept of bacterial consortia is gaining traction for treating conditions like *C. difficile* infection, IBS, and IBD. Different bacterial consortia have shown anti-inflammatory properties—depending on the bacterial species involved—and have demonstrated efficacy in reducing pro-inflammatory cytokines, vascular endothelial growth factor (VEGF), TGF-β, and matrix metalloproteinase (MMP) levels [186]. In a DSS-induced colitis mouse model, a designed bile acid consortium (BAC) composed of *Clostridium AP sp000509125*, *Bacteroides ovatus*, and *Eubacterium limosum* showed protective effects against inflammation through the production of secondary bile acids (ursodeoxycholic acid and lithocholic acid). These secondary bile acids exert protective effects by activating TGR5, which enhances gut barrier integrity and reduces inflammation [187].

The efficacy of bacterial consortia, such as a combination of *Lactobacillus reuteri*, *Lactobacillus gasseri*, *Lactobacillus acidophilus*, and *Bifidobacterium lactis*, has been compared to both placebo and single-strain treatments in DSS-induced colitis mouse models, demonstrating superior outcomes in reducing inflammation and accelerating recovery. Additionally, bacterial consortia can reshape the structure and composition of gut microbiota, reinforcing the link between gut flora and colitis phenotype [188].

Similarly, administering GUT-103, a bacterial consortium composed of 17 strains, in DSS-induced colitis mouse models, reduces pathobionts while increasing beneficial bacteria. It also decreases inflammatory cytokines and activates interleukin-10 cells, leading to reduced inflammatory responses [189].

Recent studies on the role of bacterial consortia in animal colitis models are summarized in Table 3.

The long-term application of bacterial consortia depends on their effects on the host, which are influenced by factors such as patient age, microbial characteristics, and concomitant diseases. Although some studies have reported side effects—including nausea, diarrhea, constipation, frequent bowel movements, and, less frequently, upper respiratory tract infections, as well as musculoskeletal and mediastinal disorders—treatment with bacterial consortia is generally considered safe. Careful donor screening and the selection of specific strains could further reduce the incidence of adverse effects. Additionally, bacterial consortia may influence drug efficacy, either enhancing or diminishing their therapeutic activity [191].

Bacterial consortia have demonstrated immense potential and may represent the most promising microbiota-targeting therapy. They offer a more precise and targeted modulation of the gut microbiota compared to FMT, with the added advantage of a more standardized preparation process—similar to the production of conventional pharmaceuticals. However, several challenges remain, including issues related to efficacy, safety, microorganism compatibility, host adaptation, competition with native microbes, standardization, and donor selection. Most importantly, a precise definition of the agents composing bacterial consortia is essential for their successful application.

Research on FMT may provide valuable insights in this regard. As highlighted in a recent consensus conference, an ideal FMT trial should test ‘supervised’ FMT, where donor stool is pre-screened and selected based on specific microbiome characteristics to ensure optimal composition and safety. Such a study design would offer deeper insights into the effects of specific bacterial strains on the recipient’s microbiota, guiding the selection of bacterial consortia components [192].

Given the current lack of human studies, further preclinical and clinical research is essential to validate the potential application of bacterial consortia in the context of IBD.

### 4.5. Bacteriophages

Bacteriophages are ubiquitous, self-replicating viruses that infect bacteria by interacting with bacterial receptors [193]. Bacteria have evolved various defense mechanisms against bacteriophages, including clustered regularly interspaced short palindromic repeats (CRISPR), restriction endonucleases, and other mechanisms that remain unknown [194]. The use of bacteriophages in treating multidrug-resistant (MDR) pathogens has shown promising short-term results, although bacterial resistance and host immunity remain challenges [195]. The long-term efficacy of phage therapy for gastrointestinal pathogens is still unclear. Notably, phages are currently being tested against specific bacteria associated with intestinal infections, such as *C. difficile* and *Fusobacterium nucleatum* [196,197].

Understanding the functions of gut commensal bacteria and identifying the microbes involved in dysbiosis and inflammatory bowel disease (IBD) could open new avenues for phage-based therapies. According to experimental results, gut phages may influence IBD pathogenesis through three main pathways: modulating inflammation and immune responses, regulating gut microbiota, and altering gut phage diversity [198]. Recent studies have highlighted the effectiveness of orally administered combination phage therapy in suppressing pathogenic bacteria linked to disease exacerbation. For example, the gut microbiota of four geographically distinct IBD cohorts was analyzed, revealing Klebsiella pneumoniae strains as key contributors to disease severity. Transferring these strains from IBD patients to colitis-prone, germ-free mice worsened intestinal inflammation. However, a lytic five-phage combination targeting both sensitive and resistant *K. pneumoniae* strains through distinct mechanisms successfully suppressed bacterial growth, reduced inflammation, and alleviated disease severity in colitis-prone mice [199].

Future next-generation *K. pneumoniae* phage combinations could target additional strains or even the entire species, potentially enhancing the efficacy of phage therapy against intestinal auto-inflammation. However, developing reliable phage-based treatments will require not only precise bacterial identification but also a thorough understanding of phage safety profiles, bacterial resistance mechanisms, and effective phage delivery systems.

So far, no life-threatening adverse effects have been linked to phage therapy, which generally appears to be safe [200,201]. Nevertheless, several challenges must be addressed. Bacteriophages exhibit a narrow spectrum of activity, often targeting only a few strains within a single bacterial species, which limits their utility for polymicrobial infections [202,203]. The lysogenic cycle presents another challenge, as lysogenic phages can integrate into bacterial genomes, preventing lysis and interfering with other lytic phages [204].

Standardizing phage therapy preparations remains difficult, complicating the definition of dosage, administration routes, and the evaluation of clinical efficacy [205,206]. Moreover, prolonged use of a single phage can lead to the emergence of bacteriophage-resistant strains, threatening the long-term sustainability of phage therapy. Resistance mechanisms include abortive infection, adsorption inhibition, and restriction-modification systems [202,207].

Another concern is the release of endotoxins when bacteriophages lyse their bacterial hosts, potentially exacerbating infections and increasing the risk of sepsis, as shown in preclinical studies [208]. Additionally, phages themselves can act as immunogenic agents: the release of foreign proteins from phages may trigger immune responses, including allergic reactions [209].

Despite ongoing research, studies on virome composition and its variations during IBD progression remain scarce. The clinical application of phage therapy will require overcoming these challenges by refining phage combinations, establishing regulatory frameworks, improving policies, and determining appropriate treatment doses to minimize endotoxin release.

### 4.6. Engineered Probiotics

Engineered probiotics have emerged as a novel strategy to deliver active molecules directly into the intestine, reducing drug-related side effects and evolving into promising therapeutic tools for IBD. The most common engineering techniques involve plasmid transfection. Plasmids are small, circular DNA molecules that are physically separate from chromosomal DNA and capable of autonomous replication. Due to their ability to transfer genetic material into bacterial cells, plasmids are considered essential vehicles for inducing the expression of recombinant DNA in target probiotic strains [210].

The advantage of engineered probiotics lies in their ability to selectively express anti-inflammatory molecules with localized release, increasing target site concentrations while minimizing systemic side effects [211]. Research has primarily focused on transfecting plasmids encoding anti-inflammatory cytokines, with *E. coli* strains being the preferred choice due to their genetic stability and non-transferability of plasmids pMUT1 and pMUT2 [212,213]. *Lactococcus lactis* has also shown promising results, thanks to its fully sequenced genome and versatility [214], making it easier to manage and a viable candidate for IBD treatment [215].

In a DSS-induced colitis mouse model, the administration of genetically engineered *E. coli* Nissle 1917 (EcN-Sj16) reduced clinical colitis severity, increased microbiota diversity, and enhanced butyrate production [216]. Similarly, in another DSS-treated colitis model, recombinant *E. coli* Nissle 1917 secreting interleukin-10 successfully suppressed intestinal inflammation in UC mice and protected the intestinal mucosa from injury [217].

Few studies have explored the efficacy of engineered *Lactobacillus paracasei* strains in colitis models through the release of palmitoylethanolamide (PEA), a molecule with anti-inflammatory effects [218]. Its effectiveness was further confirmed in mucosal biopsies of UC patients [219]. The administration of *L. paracasei* strains (KBL382, 384, 385) demonstrated protective effects in DSS-induced colitis models, with *L. paracasei* KBL382-treated mice showing reduced levels of pro-inflammatory cytokines, including IL-4, IL-6, TNF-α, and IL-17a [220].

Engineered *Bifidobacterium longum* has been studied for its high acid and bile tolerance, enabling survival in the gastrointestinal tract [221]. For example, administering *B. longum* expressing a PEP-1-hMnSOD fusion protein in DSS-induced colitis mice reduced inflammatory cytokines (TNF-α, IL-1β, IL-6, and IL-8) and mitigated histological damage in colonic tissues [222,223].

Engineered probiotics also include outer membrane vesicles (OMVs), nanostructures (20–300 nm) derived from the outer membrane of Gram-negative bacteria [224]. OMVs are involved in various physiological processes, facilitating intracellular factor transfer and modulating immune responses. Recently, bacterial OMVs have gained attention as promising drug delivery vehicles. In murine IBD models, OMVs derived from *E. coli* Nissle 1917 and *Bacteroides fragilis* demonstrated significant reductions in intestinal inflammation [225,226].

Recent studies on engineered probiotics are summarized in Table 4.

Recent advances in synthetic biology suggest that engineered probiotics could be used to selectively detect disease biomarkers already utilized in clinical practice. For instance, in the DSS-mice model, a bacterial promoter in the probiotic strain *E. coli* Nissle 1917 (ECN) showed increased expression in response to calprotectin. Moreover, the engineered probiotic successfully differentiated human patients with active IBD from those in remission or without IBD, using stool samples [229].

Although numerous in vivo and in vitro studies have demonstrated the potential efficacy of engineered probiotics in various animal models of IBD, significant differences between murine and human microbiota often prevent the successful translation of preclinical findings to human studies. Consequently, the current lack of robust evidence in clinical models limits their immediate application in clinical practice [230]. Some studies indicate that engineered probiotics may not transfer modified DNA to the environment, while still being capable of releasing bioactive molecules directly to target tissues, thereby reducing systemic side effects [210].

This therapeutic approach presents important challenges, including concerns about safety, specificity, bioavailability, and efficacy, alongside ethical and regulatory considerations. As innovative biological products, engineered probiotics straddle the intersection of gene therapy and personalized medicine. Regulatory barriers persist due to the need to establish clear safety and approval standards [211]. Another critical challenge is the high cost of development, driven by advanced technologies and rigorous validation processes, making engineered probiotics more expensive than traditional alternatives [231].

Finally, as with fecal microbiota transplantation (FMT), microbiota manipulation raises ethical questions about long-term safety and the risk of potential off-target effects that may compromise patient safety [232].

Despite these challenges, current findings suggest that engineered probiotics are promising candidates for the treatment of IBD.

### 4.7. Direct Metabolic Pathways Modulation and Nanotherapeutics

The alteration of metabolic pathways in IBD has emerged as one of the most promising microbial-based therapeutic strategies. Recent literature has highlighted the critical role of tryptophan and its metabolic pathways, as several tryptophan metabolism end-products are essential for maintaining intestinal homeostasis [233]. Certain metabolites modulate the activation of the aryl hydrocarbon receptor (AhR), influencing immune cell functions, promoting IL-17 and IL-22 production, and sustaining ILC3 populations in the gut [234,235,236]. A reduced output of tryptophan metabolites contributes to intestinal inflammation and impaired tissue healing, although the precise mechanisms remain unclear [237]. In patients with IBD, serum tryptophan levels are lower than in healthy controls, with a corresponding increase in the KYN/TRP ratio [238].

Several gene loci associated with the TRP–KYN metabolic pathway, including IL23R, NOD2, and ATG16L1, have been linked to IBD susceptibility and progression [239,240]. The role of metabolites from the kynurenine pathway has been extensively studied. A recent investigation in both mice and humans demonstrated a negative correlation between lower levels of xanthurenic acid (XANA) and kynurenic acid (KYNA) and the severity of intestinal inflammation. Notably, supplementation with XANA or KYNA had protective effects on intestinal epithelial cells and T cells, reducing the severity of inflammation. Additionally, the use of recombinant aminoadipate aminotransferase (AADAT), an enzyme involved in XANA and KYNA production, modulated tryptophan metabolism and conferred protective effects in DSS-induced colitis models [241]. Another study revealed an increase in serum KYNA levels, alongside a significant decrease in serum KYNA levels, regulated by the rate-limiting enzymes IDO-1 and KAT2, in DSS-induced colitis mice [242]. However, the precise mechanisms linking tryptophan metabolism to IBD remain poorly understood, and further investigations, including randomized controlled trials, are necessary to assess effectiveness and safety in human models [242].

Oxidative stress caused by excessive reactive oxygen species (ROS) production is a key factor in the initiation and progression of IBD. Nanotherapeutics represent a novel, targeted, and safer therapeutic strategy aimed at reducing oxidative stress levels. Targeting oxidative stress through molecular pathways, such as MAPK, TLR4/NF-κB, Nrf2, and PI3K/Akt—which regulate inflammation and cellular responses to oxidative damage—offers new opportunities for IBD management [243]. In DSS-induced colitis models, LBL-CO@MPDA nanotherapeutics have shown efficacy in alleviating inflammation and restoring gut barrier integrity through multiple mechanisms, including microbiota modulation, oxidative stress reduction, and immune homeostasis restoration. Specifically, the LBL-CO@MDPA nanocomposite exploits electrostatic interactions between its negatively charged surface and positively charged inflamed colon lesions, enabling targeted accumulation in inflamed tissues. The release of carbon monoxide promotes macrophage M2 polarization via the notch/Hes1/Stat3 signaling pathway while suppressing inflammation by downregulating p38 MAPK and NF-κB (p50/p65) signaling [244].

Similarly, inflamed colon-targeted antioxidant nanotherapeutics (ICANs) modulate oxidative stress in DSS-induced colitis models. ICANs consist of mesoporous silica nanoparticles (MSNs) with surface-attached ROS-scavenging ceria nanoparticles (CeNPs) and poly(acrylic acid) (PAA), which adhere to inflamed tissues via electrostatic interactions. Orally administered ICANs have demonstrated their ability to regulate the gut microenvironment by restoring redox balance, reducing inflammatory cell infiltration, and suppressing colitis-associated immune responses [245].

Despite their specificity and precision in targeting inflamed tissues, nanotherapeutics have not yet been licensed for clinical use in IBD, primarily due to challenges in translating findings from animal models to humans. The safety and toxicity profiles of these nanoparticles in human cells remain incomplete [246]. Moreover, the complex interplay of oxidative stress, immune dysregulation, and microbiota imbalances in the IBD microenvironment is not yet fully understood. Additional concerns include the low reliability and acceptability of nanotherapeutics, as well as the potential toxicity of nanoparticles. As nanotherapeutics represent an emerging research tool, further studies are essential to determine their long-term effectiveness, safety, and pharmacokinetics. 

**Figure 1:**
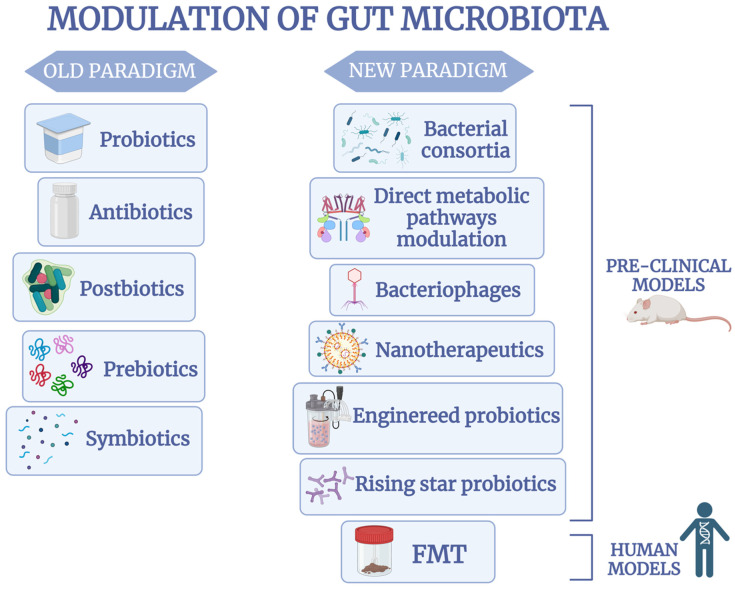
Microbial biotherapeutics as new therapeutic tools in gut microbiota modulation. The effectiveness of most new microbiota modulation therapies has been demonstrated in preclinical models and the early stages of disease in human models. Fecal microbiota transplantation has also shown effectiveness in human models, both in the early and advanced stages of disease.

The old and the new therapeutic tools for modulating the gut microbiota in IBDs are summarized in Figure 1.

## 5. Conclusions

There is strong evidence supporting the role of gut microbiota in IBD, particularly in modulating the host’s immune response and inflammation. Building on this understanding, probiotics, prebiotics, symbiotics, and postbiotics have shown promising potential, particularly in patients with UC, for inducing clinical remission, preventing relapse, and improving quality of life. However, disappointing results from other studies have limited their use, and probiotics are primarily considered an adjunct therapy alongside conventional treatments. Probiotics are generally regarded as safe, affordable, and well-tolerated, although bloating and flatulence are the most reported side effects. The wide variety of probiotic strains and inconsistent study outcomes have raised questions about their practical applicability in clinical settings.

Advances in genetic engineering have enabled the development of innovative approaches to modulate gut microbiota despite methodological variations. Fecal microbiota transplantation (FMT) is the only method that has shown promising results in human models, both in the early and advanced stages of the disease. It has demonstrated short-term efficacy, especially in ulcerative colitis (UC) models. However, major challenges remain in evaluating long-term efficacy in UC and Crohn’s disease (CD). The possibility of FMT becoming a routine clinical practice for treating inflammatory bowel diseases (IBD) is tied to issues such as donor variability and selection.

Other microbiota modulation strategies, such as next-generation probiotics, bacterial consortia, bacteriophages, pathway modulation, and nanotherapeutics, have yielded promising results in both in vitro and animal models, providing stronger scientific validation. These methods differ from older-generation techniques because they act in a more targeted manner: not only correcting dysbiosis but also directly releasing therapeutically active molecules into the gut, thereby avoiding systemic drug administration and its associated side effects. However, their application also comes with significant limitations that may hinder widespread clinical adoption.

For example, the incomplete sequencing of bacterial genomes complicates the determination of optimal bacterial species concentrations and hinders the ability to predict potential side effects. Clinical translation of these approaches has been limited, as the human microbiota differs substantially from that of animal models. Additionally, the long-term impact on host tissues and the microbial ecosystem remains poorly understood, making the long-term adaptation of the host microbiota an ongoing challenge. Potential antibiotic resistance mechanisms and the risk of exacerbating infections are additional factors that must be assessed to determine their long-term clinical applicability. Finally, manufacturing complexity and costs may limit accessibility to these therapies.

Despite these limitations, ongoing research is continuously refining these techniques, addressing safety, scalability, and regulatory concerns. The future of microbiota modulation lies in personalized, data-driven approaches that balance efficacy with minimal risk. Integrating multiomics approaches—including metagenomics, transcriptomics, and metabolomics—will enhance our ability to characterize the gut ecosystem and its interactions with the host. This will lead to the development of highly targeted treatments and move increasingly closer to the practical application of personalized medicine in IBD management.

## Figures and Tables

**Table 1:** Efficacy of probiotics in UC patients with active or quiescent colitis.

Probiotic Tested	Reference	Disease Activity (UC vs. CD)	Trial Design	Outcomes
*L. rhamnosus* NCIMB 30174, *L. plantarum* NCIMB 30173, *L. acidophilus* NCIMB 30175 and *E. faecium* NCIMB 30176	Bjarnason et al., 2019 [90]	Quiescent UC and CD	Single center, randomized, double-blind, placebo-controlled trial	No significant differences in IBD-QoL scores; reduced intestinal inflammation in UC patients
*E. coli* Nissle 1917	Park et al., 2022 [91]	Mild to moderate active UC	Multicenter, double-blind, randomized, placebo-controlled study	Prevents exacerbations of IBD-QoL scores; achieves clinical response and endoscopic remission
*L. paracasei* (A234), *L. gasseri* (A237), *L. rhamnosus* (A119), *L. acidophilus* (A118), *L. plantarum* (A138), *L. casei* (A179), *L. reuteri* (A113), *L. lactis* (A328), *B. animalis subsp lactis* (A026), *B. breve* (A055), *B. longum subsp. longum* (A027), *B. bifidum* (A058), *B. longum* subsp. *infantis* (A041) species.	Agraib et al., 2022 [92]	Mild to moderate UC	Randomized, double-blind, placebo-controlled, parallel-arms, multicenter study	Induces clinical (partial Mayo score) and biochemical remission in UC patients
*E. coli* Nissle 1917	Oh et al., 2021 [93]	Quiescent UC	Uncontrolled, observational, retrospective study	Additional administration of *E. coli* Nissle 1917 may improve UC symptoms
Kefir (*L. pentosus*, *L. brevis*, *L. plantarum*, *L. fermentum*, *L. kefiri*, and *L. lindneri*)	Yilmaz et al., 2019 [94]	Active UC and CD	single-center, prospective, open-label randomized controlled trial	Kefir consumption may modulate gut microbiota and enhance short-term quality of life
*B. breve strain Yakult*, *L. Acidophilus*	Matsuoka et al., 2018 [95]	Quiescent UC	Multicenter, randomized, placebo-controlled, double-blind parallel-group study	No effect on relapse timing
*S. faecalis* T-110, *C. butyricum* TO-A, *B. mesentericus* TO-A	Yoshimatsu et al., 2015 [96]	Quiescent UC	Randomized, double-blind, placebo-controlled study	Supports clinical remission mantainance
*L. reuteri* ATCC55730	Oliva et al., 2012 [97]	Mild to moderate active distal UC (children)	Prospective randomized, placebo-controlled study	Reduces Mayo score and histological scores; modulates mucosal cytokine expression
*S. thermophilus* BT01, *B. breve* BB02, *B. longum* * BL03, *B. infantis* * BI04, *L. acidophilus* BA05, *L. plantarum* BP06, *L. paracasei* BP07, *L. delbrueckii* subs	Tursi et al., 2010 [98]	Relapsing mild-moderate UC	Multicenter, double-blind, randomized placebo-controlled, parallel study	Decreases UCDAI scores; improves rectal bleeding; may reinduce remission after 8 weeks of treatment
*L. acidophilus*, *L. plantarum*, *L. casei*, *L. delbruecki* subspecies *bulgaricus*, *B. breve*, *B. longum*, *B. infantis*, *S. salivarius* subspecies *thermophilus*	Miele et al., 2009 [99]	Active UC (children)	Randomized, placebo-controlled, double-blind study	Maintains clinical and endoscopic remission
*Lactobacillus* GG	Zocco et al., 2006 [100]	Quiescent UC	Single center, prospective, open-label randomized trial	Maintains remission compared to mesalazine; delays UC relapse
*B. breve strain Yakult*, *B. bifidum strain Yakult*, *L. acidophillus strain*	Kato et al., 2004 [101]	Mild to moderate active UC	randomized placebo-controlled clinical trial	Reduces clinical activity index; significantly improves post-treatment endoscopic activity index and histological score

UC: ulcerative colitis. CD: Crohn’s disease. IBD-QoL: Inflammatory Bowel Disease Quality of Life Questionnaire. UCDAI: Ulcerative Colitis Disease Activity Index.

**Table 2:** Randomized controlled trials testing the role of FMT in UC.

Way of Administration	Reference	Disease Activity	Trial Design	Results Summary
FMT by colonoscopy	Deleu et al., 2024 [127]	Moderate to severe UC	Multi-centric, double-blind, sham-controlled randomized trial	Failure to achieve steroid-free clinical remission at week 8
Lyophilized oral FMT	Haifer. et al., 2022 [128]	Mild to moderate UC	Double-blind, randomized, placebo-controlled trial	Induction of clinical remission with endoscopic remission or response at week 8; maintenance of clinical, endoscopic, and histological remission at week 56
FMT by colonoscopy and enemas	Shabat et al., 2022 [129]	Moderate to severe active UC	Single, blinded, randomized, controlled trial	UC exclusion diet (UCED) leads to higher clinical remission and mucosal healing than single donor FMT, with or without diet
Encapsuled oral FMT	Crothers et al., 2021 [130]	Mild to moderate UC	single center, double-blinded, placebo-controlled, randomized control trial	Prolonged durability of FMT-induced changes in gut bacterial community structure; association between MAIT cell cytokine production and clinical response
FMT by colonoscopy and enemas	Costello et al., 2019 [131]	Mild to moderate UC	Double blind, randomized, clinical trial	A 1-week treatment with anaerobically prepared donor FMT results in higher remission rates at week 8 compared to autologous FMT
FMT by colonoscopy and enemas	Paramsothy et al., 2017 [132]	Mild to moderate UC	Multicenter, double-blind, randomized, placebo-controlled trial	Induction of clinical remission and endoscopic improvement
FMT via enema	Moayyedi et al., 2015 [133]	Mild to severe UC	Double-blind randomized controlled trial	Induction of remission in a significantly higher percentage of patients with active UC; greater microbial diversity
FMT via nasoduodenal tube	Rossen et al., 2015 [134]	Mild to moderate UC	Single-center, double-blind, placebo-controlled, randomized, proof-of-concept phase 2 trial	No significant difference in clinical and endoscopic remission between patients receiving FMT from healthy donors and those receiving autologous FMT

UC: ulcerative colitis. FMT: fecal microbiota transplantation. MAIT: mucosal-associated invariant T cells.

**Table 3:** The role of bacterial consortia tested in animal colitis models.

Bacterial Consortia	Reference	Trial Design	Results Summary
BAC (bile acid consortium, made up of *Clostridium AP sp000509125*, *Bacteroides ovatus*, and *Eubacterium limosum*)	Zhou et al., 2023 [187]	DSS-induced colitis mice model	Increases secondary bile acids (*Ursodeoxycholic acid* (UDCA) and *Lithocholic acid* (LCA)) in vitro; exerts protective effects against colitis (reduces weight loss, increases colon length, strengthens the intestinal barrier)
*Lactobacillus reuteri*, *Lactobacillus gasseri*, *Lactobacillus acidophilus* (*Lactobacillus* spp.), and *Bifidobacterium lactis* (*Bifidobacterium* spp.)	Xu et al., 2022 [188]	DSS-induced colitis mice model	Alleviates disease phenotype; restores the composition and structure of the gut microbiota
GUT 103 (strains of genera Bacteroides, *Akkermansia*, *Clostridium*, *Faecalibacterium*) and GUT108 (strains of *Clostridium*, *Intestinimonas*, *Bitterella*, *Barneseilla*)	Van der Leile et al., 2021 [189]	Immune-mediated colitis in germ free mice	GUT-103 and GUT-108: Correct the dysbiotic microbiome environment; activate IL-10-producing immune cells; reduce inflammatory responses; restore bacterial metabolic profiles to levels observed in healthy individuals’ stool samples
BMC332	Polonsky et al., 2021 [190]	DSS-induced colitis in mice	Exhibits anti-inflammatory properties; maintains intestinal barrier integrity

DSS: dextran sulfate sodium. BAC: bile acid consortium. UDCA: ursodeoxycholic acid. LCA: lithocholic acid.

**Table 4:** Summary of recent studies on engineered probiotics.

Engineered Probiotic	Reference	Trial Design	Results Summary
*E. coli*	Wang et al., 2021 [216]	DSS-induced colitis in mice	- Improvement in colitis by the modulation of microbiota composition (protein Sj16)
*E. coli*	Cui et al., 2021 [217]	DSS-treated colitis in mice	- Suppression of intestinal inflammatory response (IL-10) - Protection of mucosa against injury
*L. paracasei* (KBL382,384,385)	Kim et al., 2019 [220]	DSS-induced colitis in mice	- Strong protective effects; improved colitis symptoms
*B. longum*	Liu et al., 2016 [222]	DSS-induced colitis in mice	- Reduction of inflammation (TNF-α, IL-1β, IL-6, and IL-8)
*B. longum*	Wei et al., 2016 [223]	DSS-induced colitis in mice	- Expression of α-MSH and significant anti-inflammatory effect
OMV of *Bacteroides thetaiotaomicron* (Bt)	Carvalho et al., 2019 [225]	DSS-induced colitis in mice	- KGF-2 reduced macroscopic and microscopic inflammation
OMV from *E. coli* Nissle 1917	Fabrega et al., 2017 [226]	DSS-induced colitis in mice	- Intestinal anti-inflammatory effects
*E. coli*	Zhang et al., 2018 [227]	DSS-induced experimental colitis in mice	- Reduction of inflammation (IL-35)
*L. lactis*	Hanson et al., 2014 [228]	DSS-induced colitis in mice	- Reduction of inflammation (IL-10)

DSS: dextran sulfate sodium. IL: interleukin. KGF-2: keratinocyte growth factor-2.

## Data Availability

Not applicable.
